# Genome‐wide identification of urinary cell‐free microRNAs for non‐invasive detection of bladder cancer

**DOI:** 10.1111/jcmm.13487

**Published:** 2018-01-24

**Authors:** Jaroslav Juracek, Barbora Peltanova, Jan Dolezel, Michal Fedorko, Dalibor Pacik, Lenka Radova, Petra Vesela, Marek Svoboda, Ondrej Slaby, Michal Stanik

**Affiliations:** ^1^ Central European Institute of Technology Masaryk University Brno Czech Republic; ^2^ Department of Urologic Oncology Masaryk Memorial Cancer Institute Brno Czech Republic; ^3^ Department of Urology University Hospital Brno Masaryk University Brno Brno Czech Republic; ^4^ Department of Comprehensive Cancer Care Masaryk Memorial Cancer Institute Brno Czech Republic

**Keywords:** bladder cancer, cell‐free miRNAs, urine, biomarker, non‐invasive diagnosis

## Abstract

Urinary microRNAs (miRNAs) are emerging as clinically useful tool for early and non‐invasive detection of various types of cancer including bladder cancer (BCA). In this study, 205 patients with BCA and 99 healthy controls were prospectively enrolled. Expression profiles of urinary miRNAs were obtained using Affymetrix miRNA microarrays (2578 miRNAs) and candidate miRNAs further validated in independent cohorts using qRT‐PCR. Whole‐genome profiling identified 76 miRNAs with significantly different concentrations in urine of BCA compared to controls (*P* < 0.01). In the training and independent validation phase of the study, miR‐31‐5p, miR‐93‐5p and miR‐191‐5p were confirmed to have significantly higher levels in urine of patients with BCA in comparison with controls (*P* < 0.01). We further established 2‐miRNA‐based urinary DxScore (miR‐93‐5p, miR‐31‐5p) enabling sensitive BCA detection with AUC being 0.84 and 0.81 in the training and validation phase, respectively. Moreover, DxScore significantly differed in the various histopathological subgroups of BCA and decreased post‐operatively. In conclusion, we identified and independently validated cell‐free urinary miRNAs as promising biomarkers enabling non‐invasive detection of BCA.

## Introduction

BCA is the most common cancer of the urinary tract, with approximately 430,000 new cases diagnosed in 2012 worldwide 1. More than 90% of BCA are urothelial carcinoma, of which around 80% are non‐muscle‐invasive (superficial, NMIBC) tumours. Superficial tumours frequently recur (50–70%) and can progress to potentially lethal muscle‐invasive form (MIBC, 10–15%) 1. Patients with BCA are diagnosed and monitored by urethrocystoscopy and may undergo multiple resections because of the long‐term follow‐up. Urethrocystoscopy as a standard diagnostic method is expensive, invasive and in case of carcinoma *in situ* (CIS) or small papillary tumours, does not exhibit sufficient sensitivity 2. A non‐invasive tool for BCA diagnostics is urine cytology; however, this method suffers from low sensitivity for the detection of low‐grade and early BCA 3. In addition, several urine‐based tests for BCA 4 have been approved for clinical use, but are not suitable because of their low specificity. Thus, new both specific and sensitive non‐invasive diagnostic biomarkers are needed for BCA detection and monitoring.

miRNAs are regulatory short non‐coding RNAs involved in the pathogenesis of wide range of cancers including BCA 5. Recently, numerous studies have proved that circulating cell‐free miRNAs exist in various body fluids, such as urine 6. Urinary miRNAs in particular present emerging novel class of molecular biomarkers because of their remarkably high stability and good analytical properties 7. In this study, we aimed to identify the cell‐free miRNA biomarkers in the urine for non‐invasive detection of BCA by use of genomewide approach in patients with BCA and age‐/gender matched healthy controls, and to validate the results in independent cohorts.

## Materials and methods

### Patients and controls

Between September 2013 and May 2016, adults undergoing endoscopic treatment with transurethral resection or radical cystectomy for known or suspected BCA and healthy controls were recruited for participation in this prospective observational study at Department of Urologic Oncology, Masaryk Memorial Cancer Institute (MMCI). The study has been approved by ethical committee of MMCI and all participating individuals signed informed consent. Healthy controls included patients surgically treated for benign urological conditions. Patients with active malignancy or history of any cancer, urinary tract infection, and foreign bodies in urinary tract or urolithiasis were excluded from control group. Urine samples of the patients were collected prior to surgery. In 13 disease‐free NMIBC patients, we collected urine also post‐operatively, 3 months after surgery. In one case, urine was collected during the whole follow‐up to enable analysis of the dynamics of identified biomarkers.

The median age of the 205 patients with BCA (154 males, 51 females) and 99 HC (71 males, 28 females) enrolled were 68 (range 31–86) and 66 (range 51–80) years, respectively. There were 68 cases of low‐grade and 137 cases of high‐grade tumours included in the study. NMIBC (Ta, T1, Tis) and MIBC (T2‐4) patients were represented by 140 and 65 cases, respectively. Clinical‐pathological characteristics of the cohorts are summarized in Table S1. We further enrolled 30 patients (20 males, 10 females) with non‐metastatic clear‐cell renal cell carcinoma (ccRCC) with median age 66 (range 33–87) years as a control cohort to evaluate specificity of the identified biomarkers for BCA.

### Sample processing and RNA isolation

Samples of first morning voided urine were collected in 15 ml tubes with EDTA used for nucleic acid preservation, centrifuged at 4°C at 2000 *g* for 15 min. Supernatant was collected and stored at ‐80°C until analysed. Urine samples were centrifuged again before RNA isolation at 4°C at 12,000 g for 15 min. Total RNA from 1 ml of cell‐free urine supernatant was isolated using Urine microRNA Purification Kit (Norgen Biotek, Thorold, ON, Canada). Tumour tissue and adjacent bladder non‐tumour tissue were removed within transurethral resection of tumour or radical cystectomy, placed in in RNAlater™ Stabilization Solution (Invitrogen by Thermo Fisher Scientific, Waltham, MA, USA) and stored at −80°C until analysed. Total RNA enriched for fraction of small RNA was isolated using mirVana™ miRNA isolation kit (Invitrogen by Thermo Fisher Scientific, Waltham, MA, USA) according to manufacturer's recommendations. Quality and quantity of RNA were determined using the NanoDrop™ 2000 spectrophotometer (Thermo Fisher Scientific, Waltham, MA, USA).

### Whole‐genome microRNA profiling (discovery phase)

In the discovery phase of the study, the cell‐free urine supernatant of 15 patients with BCA and 16 HC was analysed using GeneChip miRNA 3.0 arrays (Affymetrix by Thermo Fisher Scientific, Waltham, MA, USA) enabling detection of 2578 human mature miRNAs. The GeneChip raw data were normalized and statistically evaluated in the environment of statistical language R 8 using the Bioconductor package concerning miRNA profiling combined with hierarchical clustering. miRNAs meeting the pre‐defined selection criteria (fold change > 2.5, average signal > 3, *P* < 0.0025) were forwarded to the training phase of the study.

### Quantitative reverse transcription PCR (training and validation phase)

For validation of microRNA profiling data, concentrations of miR‐31‐5p, miR‐93‐5p and miR‐191‐5p were determined in the urinary samples of 140 patients with BCA, 67 HC and 30 RCC patients (training phase) and 50 patients with BCA and 16 HC (validation phase) by use of quantitative reverse transcription PCR (qRT‐PCR) accordingly to the standard TaqMan MicroRNA Assay protocol (miR‐31‐5p: ID002279, miR‐93‐5p: ID001090, miR‐191‐5p: ID002299; Thermo Fisher Scientific) on Roche LightCycler 480 PCR system. We included interplate calibrator on each plate for each assay enabling us to correct for interplate variability. Quantitatively, all measurements were standardized by use of the same amount of total RNA entering the reverse transcription and PCR reaction. Regarding absolute quantification approach, chemically synthesized miRNA oligos (IDT, Coralville, IA, USA) were serially diluted and carried out in parallel with qRT‐PCR of biological samples. Ct values of biological samples were converted to absolute concentration of miRNAs in the cell‐free supernatant of the urine (fmol/l) based on relevant calibration curve equation. For all qRT‐PCR measurements, we used interplate controls and non‐template negative controls to enable interplate comparisons and eliminate contaminations.

### Statistical analysis

Differences in microRNA level between compared cohorts were evaluated by Mann–Whitney U‐test and Kruskal–Wallis test. The ROC (receiver operating characteristic) analysis was performed to identify the optimal cut‐off value enabling discrimination of patients with BCA and HC. Bidirectional stepwise logistic regression model was used to establish the combined miRNA diagnostic score. Statistical analysis was performed with GraphPad Prism version 6.00 for Windows (GraphPad Software, La Jolla, CA, USA, www.graphpad.com). *P* < 0.05 was considered to indicate a statistically significant difference.

## Results

Using whole‐genome profiling, we identified 76 miRNAs with significantly different levels in the urine of patients with BCA compared to the urine of HC (*P* < 0.01), thereof 64 had higher and 12 had lower levels in the urine of patients with BCA (Table S2, Fig. S1). Genomewide microRNA expression data have been deposited in the ArrayExpress database at EMBL‐EBI (www.ebi.ac.uk/arrayexpress) under accession number E‐MTAB‐6102. Based on the discovery phase data and using pre‐defined selection criteria, we selected miR‐31‐5p, miR‐93‐5p and miR‐191‐5p for further independent validation.

**Figure 1:**
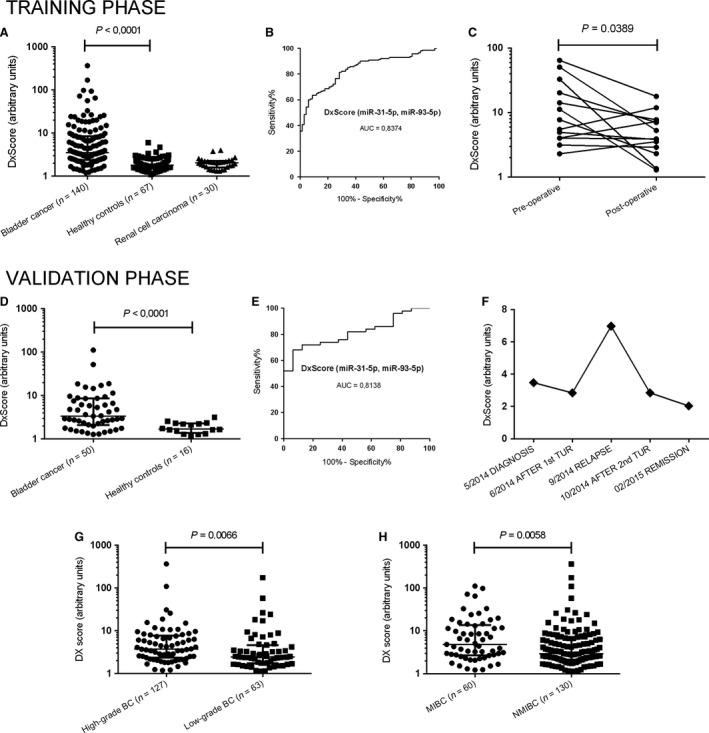
Analytical characteristics of diagnostic scoring system (DxScore) based on the combination of cell‐free miR‐31‐5p and miR‐93‐5p concentrations in the urine supernatant. (**A**) Training phase – DxScore in bladder cancer patients, healthy controls and renal cell carcinoma patients (ANOVA (Kruskal‐Wallis test), *P* < 0.0001). (**B**) Training phase – ROC analysis of DxScore to evaluate the ability to distinguish patients with bladder cancer and healthy controls (*P* < 0.0001; AUC = 0.84; sensitivity 82%. specificity 70%). (**C**) Significant decrease in DxScore in urine samples collected 3 months after tumour resection in comparison with pre‐operative samples (Wilcoxon Rank Sum Test; *P* = 0.04). (**D**) Validation phase – DxScore in patients with bladder cancer and healthy controls (Mann–Whitney U‐test, *P* < 0.0001). (**E**) Validation phase – ROC analysis of DxScore to validate the ability to distinguish patients with bladder cancer and healthy controls using cut‐off value from training phase (*P* < 0.0001; AUC = 0.81; sensitivity 74%. specificity 75%). (**F**) Dynamics of DxScore within follow‐up of patient with bladder cancer developing recurrence of the disease. (**G**) DxScore in low‐grade and high‐grade NMIBC cases (Mann–Whitney U‐test; *P* = 0.0066). (**H**) DxScore in MIBC and NMIBC patients (Mann–Whitney U‐test; *P* = 0.0058).

Within training phase of the study, we determined expression level of miR‐31‐5p, miR‐93‐5p and miR‐191‐5p in urine samples of 140 patients with BCA, 67 HC and 30 RCC patients. The concentrations of miR‐31‐5p (*P* < 0.0001), miR‐93‐5p (*P* < 0.0001) and miR‐191‐5p (*P* < 0.0001) in the urine of patients with BCA were significantly higher in comparison with both control groups, healthy controls and patients with RCC (Table 1, Fig. S2A). Subsequent ROC analysis reflects ability of miR‐31‐5p, miR‐93‐5p and miR‐191‐5p to distinguish between BCA cases and controls with AUC being 0.78, 0.8 and 0.76, respectively (Fig. S2B). Further, we used logistic regression to establish diagnostic score (DxScore) combining expression of miR‐31‐5p, miR‐93‐5p and miR‐191‐5p. DxScore enables differentiate BCA and HC with the sensitivity of 81% and specificity of 70% (AUC = 0.83, *P* < 0.0001). In the logistic regression, miR‐191‐5p was not statistically significant and independent factor, therefore, we excluded this miRNA from further analysis and reduced the number of candidate diagnostic miRNAs from 3 to 2 that were subsequently included in the final 2‐miRNA‐based DxScore = 1.0349‐1.7308*miR‐31‐5p‐1.1144*miR‐93‐5p (cut‐off = 2.2). ROC (receiver operating characteristic) analysis proved that usage of this 2‐miRNA‐based urinary DxScore enables to distinguish between patients with BCA from HC with the sensitivity of 82% and specificity of 70% and AUC being 0.84 (Table 1, Fig. 1A and B).

**Table 1:** Analytical performance of miRNA and combined DxScores in training and validation phase of the study

miRNA	Training phase					Validation phase				
	Bladder cancer *versus* healthy controls^a^	Fold change	AUC (Cut‐off value)	Sensitivity	Specificity	Bladder cancer *versus* healthy controls^a^	Fold change	AUC (Cut‐off value)	Sensitivity	Specificity
miR‐31‐5p	<0.0001	4.9	0.78 (0.6)	74	73	0.0009	4.8	0.77	75	68
miR‐93‐5p	<0.0001	33.1	0.80 (0.2)	74	72	<0.0001	33.8	0.83	68	87
miR‐191‐5p	<0.0001	9.1	0.76 (0.3)	73	68	0.0061	7.6	0.73	74	50
DxScore (miR‐31‐5p, miR‐93‐5p, miR‐191‐5p)	<0.0001	5.7	0.83 (2.3)	81	70	0.0002	4.2	0.80	74	70
DxScore (miR‐31‐5p, miR‐93‐5p)	<0.0001	6.1	0.84 (2.2)	82	70	<0.0001	4.5	0.81	74	75

a *P*‐value (Mann–Whitney test); AUC, area under curve.

On independent cohort of 50 BCA cases and 16 HC, we confirmed significantly higher expression levels of miR‐31‐5p, miR‐93‐5p and miR‐191‐5p in patients with BCA (*P* = 0.0009, *P* < 0.0001 and *P* = 0.0061 resp., Fig. S2C and D). In the same cohort, we validated analytical performance of 2‐miRNA‐based DxScore using cut‐off value obtained within training phase of the study. DxScore was able to differentiate patients with BCA and HC with the sensitivity of 74% and specificity of 75% and AUC being 0.81 (Fig. 1D and E). Moreover, DxScore allowed to discriminate low‐grade and high‐grade NMIBC cases (*P* = 0.0066, Fig. 1G) and MIBC and NMIBC (*P* = 0.0058, Fig. 1H). In addition, we observed significant decrease in miR‐31‐5p, miR‐93‐5p and miR‐191‐5p concentrations (Fig. S2E) and 2‐miRNA‐based DxScore in the urinary samples collected 3 months after surgery in disease‐free patients compared to pre‐operative samples (Fig. 1C). In one NMIBC case with recurrence and available set of follow‐up urinary samples, we have shown that miR‐31‐5p, miR‐93‐5p and miR‐191‐5p (Fig. S2F) and 2‐miRNA‐based DxScore follows the status of the disease (Fig. 1F).

In BCA tumour tissue samples, we observed significantly higher expression levels of miR‐93‐5p and miR‐191‐5p (*P* = 0.002, fold change = 2.6 and *P* = 0.002, fold change = 2.5, resp.) in comparison with control non‐tumour bladder tissues (Fig. S3).

## Discussion

Urinary cell‐free miRNAs are emerging as potential biomarkers of urologic cancers 7. In our previous study, we have shown that cell‐free miRNAs are abundant in urine of renal cell carcinoma patients and present promising non‐invasive biomarkers 9. Similarly, several reports described specific miRNA expression profiles in urine of patients with BCA indicating their potential usage for early detection of the disease, prognosis prediction or therapy response 10, 11, 12. However, there is a low overlap in identified miRNAs in independent studies. Majority of the studies published so far were statistically underpowered with low number of patients or with the absent independent validation cohort. Moreover, these studies suffer either with small analytical performance 13 or with diagnostic/prediction systems based on expression of large number of miRNAs 14 and differed in important technological aspects, mainly linked to the pre‐analytical phase of the analysis, which can largely affect obtained results 15, 16, 17.

In our study, we identified and validated miR‐31‐5p, miR‐93‐5p and miR‐191‐5p to have significantly higher concentrations in urine of patients with BCA in comparison with controls. Concentration of miR‐93‐5p and miR‐191‐5p was significantly elevated also in tumour tissue compared to non‐tumour urothelium, which may be associated with increased secretion of these miRNAs by BCA cells into the urine. Based on the TCGA BCA dataset, miR‐93‐5p is significantly up‐regulated in tumour tissue in comparison with non‐tumour urothelium and miR‐31‐5p is the most significantly deregulated miRNA defining class 4 BCA 18.

We further established and independently validated 2‐miRNA‐based urinary DxScore (miR‐93‐5p, miR‐31‐5p) enabling sensitive BCA detection. MiR‐191‐5p was omitted because of lowest statistical significance and other monitored outputs (AUC, specificity and sensitivity). Urquidi *et al*. presented diagnostic model predicting the presence of BCA with high sensitivity and specificity 14, but based on the combination of 25 miRNA levels. We believe that reduced number of biomarkers would be a great advantage for possible clinical application of such diagnostic score. In addition to BCA detection, our DxScore enabled also to distinguish between NMIBC and MIBC and low‐grade and high‐grade form of NMIBC and significantly decreased in the urinary samples collected after surgery in disease‐free patients compared to pre‐operative samples. When compared to previous studies 13, 17, our study is performed on the larger cohort of well‐defined patients with BCA and healthy controls, showing more promising analytical characteristics of cell‐free urinary miRNAs in BCA than described before.

To conclude, our study is clearly showing the diagnostic potential of urinary cell‐free miRNAs, but further independent studies are needed to confirm our results and prove potential clinical utility of our 2‐miRNA‐based DxScore for non‐invasive diagnosis and monitoring of BCA.

## Conflict of interests

No potential conflict of interests were disclosed.

## Supporting information


**Figure S1**. Hierarchical clustergram discriminating bladder cancer patients and healthy controls according to differentially expressed miRNAs (blue color indicates healthy controls; yellow color indicates patients; *P* < 0.01).

**Figure S2.** Analytical characteristics of miR‐31‐5p, miR‐93‐5p and miR‐191‐5p.

**Figure S3.** Concentrations of miR‐31‐5p, miR‐93‐5p and miR‐191‐5p in BCA tumor tissue and adjacent bladder non‐tumor tissue (*P* = 0.1309, fold change = 2.3; *P* = 0.002, fold change = 2.6 and *P* = 0.002, fold change = 2.5 resp).

**Table S1.** Clinicopathological characteristics of study subjects.
**Table S2**. MicroRNAs with different levels in the urine of bladder cancer patients compared with healthy controls (76 miRNAs with *P* < 0.01).
