# Supplementary figures and images for: Genome‐wide identification of urinary cell‐free microRNAs for non‐invasive detection of bladder cancer
Source: J Cell Mol Med. 2018 Jan 24;22(3):2033–8. doi: 10.1111/jcmm.13487 (PMC5824364; doi:10.1111/jcmm.13487)

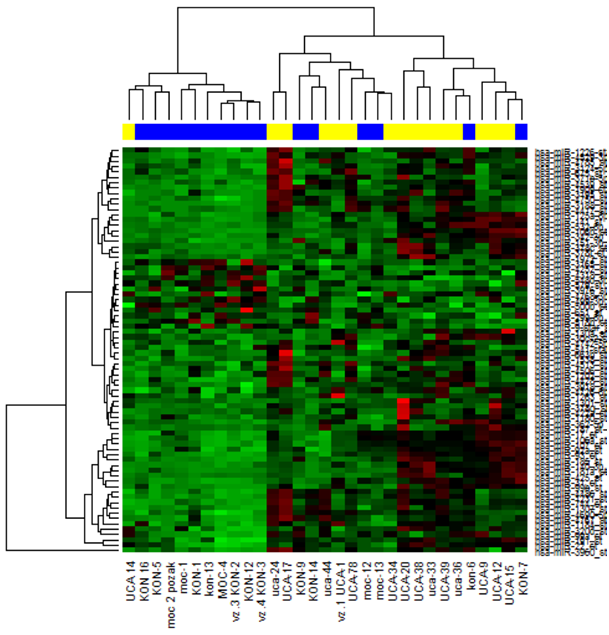

Supplement: Supplementary file 1 — Figure S1. Hierarchical clustergram discriminating bladder cancer patients and healthy controls according to differentially expressed miRNAs (blue color indicates healthy controls; yellow color indicates patients; P < 0.01). [file JCMM-22-2033-s001.tif]
