# Supplementary material for: Genome‐wide identification of urinary cell‐free microRNAs for non‐invasive detection of bladder cancer
Source: J Cell Mol Med. 2018 Jan 24;22(3):2033–8. doi: 10.1111/jcmm.13487 (PMC5824364; doi:10.1111/jcmm.13487)
Supplement: Supplementary file 4 — Table S1. Clinicopathological characteristics of study subjects. Table S2. MicroRNAs with different levels in the urine of bladder cancer patients compared with healthy controls (76 miRNAs with P < 0.01). [file JCMM-22-2033-s004.docx]

**SUPPLEMENTARY DATA**

**Genome-wide identification of urinary cell-free microRNAs for non-invasive detection of bladder cancer**

Jaroslav Juracek^1^, Barbora Peltanova^1^, Jan Dolezel^2^, Michal Fedorko^3^, Dalibor Pacik^3^, Lenka Radova^1^, Marek Svoboda^4^, Ondrej Slaby^1.4*^, Michal Stanik^2*^

**Supplementary Figure Legends**

Supplemetary Figure S1 Hierarchical clustergram discriminating bladder cancer patients and healthy controls according to differentially expressed miRNAs (blue color indicates healthy controls; yellow color indicates patients; P < 0.01).

Supplemetary Figure S2 Analytical characteristics of miR-31-5p. miR-93-5p and miR-191-5p.

(A) Training phase - Concentrations of miR-31-5p, miR-93-5p and miR-191-5p in bladder cancer patients, healthy controls and renal cell carcinoma patients (ANOVA, P<0.0001, P<0.0001, P<0.0001 resp.). (B) Training phase - ROC analysis of miR-31-5p, miR-93-5p and miR-191-5p to evaluate the ability to distinguish bladder cancer patients and healthy controls (P<0.0001; AUC=0.78; sensitivity, 74%. specificity 73%; P<0.0001; AUC=0.80; sensitivity 74%, specificity 72%; P<0.0001; AUC=0.76; sensitivity 73%, specificity 68% resp.). (C) Validation phase - Concentrations of miR-31-5p, miR-93-5p and miR-191-5p in bladder cancer patients and healthy controls (Mann-Whitney U test; P=0.0009, P<0.0001, P=0.0061 resp). (D) Validation phase - ROC analysis of miR-31-5p, miR-93-5p and miR-191-5p to validate the ability to distinguish bladder cancer patients and healthy controls using cut-off value from training phase (P=0.001; AUC=0.77; sensitivity, 75%. specificity 68%; P<0.0001; AUC=0.83; sensitivity 68%, specificity 87%; P=0.007; AUC=0.73; sensitivity 74%, specificity 50% resp.). (E) Decrease of miR-31-5p, miR-93-5p and miR-191-5p level in urine samples collected 3 months after tumor resection in comparison with pre-operative samples (Wilcoxon Rank Sum Test; P=0.0803, P=0.0105, P=0.0046 resp.). (F) Dynamics of miR-31-5p, miR-93-5p and miR-191-5p levels within follow-up of bladder cancer patient developing recurrence of the disease.

Supplementary Figure S3 Concentrations of miR-31-5p, miR-93-5p and miR-191-5p in BCA tumor tissue and adjacent bladder non-tumor tissue (P=0.1309, fold change=2.3 ; P=0.002, fold change=2.6 and P=0.002, fold change=2.5 resp.).

**Supplementary Tables**

Supplementary Table S1 Clinicopathological characteristics of study subjects.

| **Characteristics** | **Discovery phase** | **Training phase** | **Validation phase** |
| --- | --- | --- | --- |
| **Bladder cancer cases** | | | |
| **Number** | 15 | 140 | 50 |
| **Age (mean ± s.d.)*, years** | 68.4 ± 10.7 | 66.8 ± 9.9 | 68.7 ± 9.2 |
| **Sex, number (%)** | | | |
| Male | 9 (60) | 110 (79) | 35 (70) |
| Female | 6 (40) | 30 (21) | 15 (30) |
| **Grade, number (%)** | | | |
| Low-grade | 5 (33) | 45 (32) | 18 (36) |
| High-grade | 10 (67) | 95 (68) | 32 (64) |
| **TNM stage, number (%)** | | | |
| Tis | 0 (0) | 3 (2.1) | 5 (15) |
| Ta | 9 (60) | 53 (38) | 17 (33) |
| T1 | 1 (7) | 42 (30) | 10 (27) |
| T2-4 | 5 (33) | 42 (30) | 18 (25) |
| **Healthy donors** |  |  |  |
| **Number** | 16 | 67 | 16 |
| **Age (mean ± s.d.), years** | 66.4 ± 7 | 65.4 ± 9 | 64.9 ± 13 |
| **Sex, number (%)** |  |  |  |
| Male | 11 (69) | 49 (73) | 11 (69) |
| Female | 5 (31) | 18 (27) | 5 (31) |

* s.d. – standard deviation

Supplementary Table S2 MicroRNAs with different levels in the urine of bladder cancer patients compared with healthy controls (76 miRNAs with P<0.01).

|  | **Fold change** | **Average expression** | **P-value** |
| --- | --- | --- | --- |
| miR-99b | 3.80 | 2.50 | 0.0001 |
| miR-140-3p | 4.21 | 2.25 | 0.0002 |
| miR-1180 | 1.76 | 1.31 | 0.0003 |
| miR-532-5p | 2.53 | 1.71 | 0.0004 |
| miR-4529-3p | 1.94 | 1.55 | 0.0004 |
| **miR-93** | **5.15** | **3.12** | **0.0006** |
| miR-4742-5p | 1.33 | 1.01 | 0.0006 |
| miR-362-5p | 1.86 | 1.34 | 0.0006 |
| miR-1307 | 2.91 | 2.43 | 0.0006 |
| miR-299-5p | 0.81 | 1.01 | 0.0007 |
| miR-342-3p | 2.42 | 1.83 | 0.0007 |
| miR-22 | 5.92 | 4.37 | 0.0007 |
| miR-744 | 2.83 | 2.51 | 0.0008 |
| miR-141 | 2.99 | 1.93 | 0.0009 |
| miR-1184 | 2.61 | 2.38 | 0.0011 |
| miR-3188 | 2.58 | 2.33 | 0.0012 |
| miR-1231 | 3.81 | 2.55 | 0.0013 |
| miR-378i | 2.14 | 1.68 | 0.0013 |
| miR-4428 | 1.50 | 1.14 | 0.0014 |
| miR-3918 | 1.37 | 1.02 | 0.0016 |
| miR-371b-5p | 1.79 | 1.58 | 0.0016 |
| miR-500b | 0.83 | 0.71 | 0.0017 |
| miR-663b | 1.46 | 0.94 | 0.0017 |
| miR-125a-5p | 2.61 | 2.07 | 0.0018 |
| **miR-191** | **5.03** | **4.52** | **0.0019** |
| miR-1909-star | 2.11 | 2.07 | 0.0020 |
| miR-4486 | 3.45 | 3.08 | 0.0023 |
| **miR-31** | **3.63** | **2.68** | **0.0023** |
| miR-4417 | 2.91 | 2.14 | 0.0026 |
| miR-4690-5p | 2.67 | 3.10 | 0.0029 |
| miR-181a | 3.19 | 2.15 | 0.0030 |
| miR-4539 | 1.99 | 2.17 | 0.0030 |
| miR-4252 | 0.82 | 0.81 | 0.0032 |
| miR-1263 | 1.47 | 1.41 | 0.0033 |
| miR-4767 | 2.20 | 2.50 | 0.0033 |
| miR-3124-5p | 2.04 | 1.98 | 0.0034 |
| miR-4785 | 1.68 | 1.49 | 0.0034 |
| miR-4506 | 1.41 | 1.21 | 0.0035 |
| miR-579 | 0.85 | 0.97 | 0.0039 |
| miR-99a | 3.03 | 2.84 | 0.0040 |
| miR-4750 | 2.68 | 2.86 | 0.0040 |
| miR-107 | 3.61 | 2.77 | 0.0042 |
| miR-1226-star | 1.61 | 1.69 | 0.0042 |
| miR-320d | 2.97 | 3.27 | 0.0043 |
| miR-378g | 1.37 | 1.02 | 0.0045 |
| miR-187 | 1.76 | 1.16 | 0.0045 |
| miR-106a | 3.33 | 2.77 | 0.0045 |
| miR-518b | 0.82 | 0.86 | 0.0047 |
| miR-3976 | 0.85 | 0.91 | 0.0048 |
| miR-1972 | 1.97 | 1.69 | 0.0052 |
| miR-185 | 2.96 | 2.71 | 0.0053 |
| miR-1538 | 1.35 | 0.89 | 0.0054 |
| miR-675 | 1.95 | 1.88 | 0.0055 |
| miR-1301 | 1.48 | 0.95 | 0.0055 |
| miR-4768-3p | 1.20 | 0.77 | 0.0056 |
| miR-541-star | 1.20 | 0.97 | 0.0060 |
| miR-3195 | 2.06 | 1.72 | 0.0062 |
| miR-4726-5p | 1.57 | 1.51 | 0.0063 |
| miR-106b | 2.46 | 1.80 | 0.0063 |
| miR-378c | 2.12 | 1.78 | 0.0064 |
| miR-651 | 0.81 | 0.84 | 0.0067 |
| miR-17 | 3.28 | 2.88 | 0.0069 |
| miR-422a | 1.58 | 1.17 | 0.0071 |
| miR-151-3p | 2.14 | 2.03 | 0.0073 |
| miR-3165 | 0.85 | 0.82 | 0.0074 |
| miR-30c-2-star | 1.31 | 1.02 | 0.0076 |
| miR-548d-3p | 0.85 | 0.78 | 0.0081 |
| miR-92a | 2.86 | 3.26 | 0.0085 |
| miR-425 | 2.70 | 2.06 | 0.0086 |
| miR-9-star | 0.81 | 1.02 | 0.0090 |
| miR-1202 | 1.45 | 1.43 | 0.0091 |
| miR-499-3p | 0.85 | 0.78 | 0.0094 |
| miR-3960 | 1.54 | 11.19 | 0.0094 |
| miR-130a | 1.30 | 1.03 | 0.0096 |
| miR-4513 | 1.32 | 1.33 | 0.0098 |
| miR-4318 | 0.85 | 0.75 | 0.0099 |
